# Loss of KEAP1 Causes an Accumulation of Nondegradative Organelles

**DOI:** 10.3390/antiox11071398

**Published:** 2022-07-19

**Authors:** Elisabet Uribe-Carretero, Guadalupe Martinez-Chacón, Sokhna M. S. Yakhine-Diop, Gema Duque-González, Mario Rodríguez-Arribas, Eva Alegre-Cortés, Marta Paredes-Barquero, Saray Canales-Cortés, Elisa Pizarro-Estrella, Antonio Cuadrado, Rosa Ana González-Polo, José M. Fuentes, Mireia Niso-Santano

**Affiliations:** 1 Departamento de Bioquímica y Biología Molecular y Genética, Facultad de Enfermería y Terapia Ocupacional, Universidad de Extremadura, 10003 Cáceres, Spain; euribec@unex.es (E.U.-C.); gmartinezchacon@hotmail.com (G.M.-C.); geduqueg@unex.es (G.D.-G.); mariora@unex.es (M.R.-A.); evalegrec@unex.es (E.A.-C.); martapb@unex.es (M.P.-B.); sacanalesc@unex.es (S.C.-C.); osyris_tng@hotmail.com (E.P.-E.); 2 Centro de Investigación Biomédica en Red en Enfermedades Neurodegenerativas (CIBERNED), 28029 Madrid, Spain; smsyakhinediop@unex.es (S.M.S.Y.-D.); antonio.cuadrado@uam.es (A.C.); 3 Instituto Universitario de Investigación Biosanitaria de Extremadura (INUBE), 10003 Cáceres, Spain; 4 Departamento de Bioquímica, Faculdad de Medicina, Universidad Autónoma de Madrid (UAM), 28049 Madrid, Spain; 5 Instituto de Investigaciones Biomédicas Alberto Sols (CSIC-UAM), 28029 Madrid, Spain; 6 Instituto de Investigación Sanitaria La Paz (IdiPaz), 28029 Madrid, Spain

**Keywords:** autophagy, cathepsin D, endosomes, KEAP1, LAMP1, lysosomes

## Abstract

KEAP1 is a cytoplasmic protein that functions as an adaptor for the Cullin-3-based ubiquitin E3 ligase system, which regulates the degradation of many proteins, including NFE2L2/NRF2 and p62/SQSTM1. Loss of KEAP1 leads to an accumulation of protein ubiquitin aggregates and defective autophagy. To better understand the role of KEAP1 in the degradation machinery, we investigated whether Keap1 deficiency affects the endosome-lysosomal pathway. We used KEAP1-deficient mouse embryonic fibroblasts (MEFs) and combined Western blot analysis and fluorescence microscopy with fluorometric and pulse chase assays to analyze the levels of lysosomal-endosomal proteins, lysosomal function, and autophagy activity. We found that the loss of keap1 downregulated the protein levels and activity of the cathepsin D enzyme. Moreover, KEAP1 deficiency caused lysosomal alterations accompanied by an accumulation of autophagosomes. Our study demonstrates that KEAP1 deficiency increases nondegradative lysosomes and identifies a new role for KEAP1 in lysosomal function that may have therapeutic implications.

## 1. Introduction

Kelch-like ECH-associated protein 1 (KEAP1) is a cytoplasmic protein that acts as an adaptor molecule for the Cullin-3-based ubiquitin E3 ligases, which target several proteins for degradation [1,2,3,4]. KEAP1 is the main negative regulator of the transcription factor nuclear factor erythroid 2 p45-related factor (NRF2, gene name *NFE2L2*), a transcription factor that regulates cellular defense and survival pathways [5]. Under normal conditions, KEAP1 inhibits NRF2 activity by promoting its ubiquitination and proteasomal degradation. However, under stress conditions, KEAP1 is inactivated and nascent NRF2 can translocate into the nucleus and promote the induction of cytoprotective genes [6]. In addition to interacting with NRF2, KEAP1 contains several distinct binding domains allowing its interaction with several proteins [7]. For instance, an interaction between KEAP1 and p62, an autophagy-related protein that connects KEAP1 to autophagy, has been described [8]. In fact, KEAP1 is reduced by autophagy and the loss of KEAP1 leads to an accumulation of protein ubiquitin aggregates and defects in autophagy [9].

Many studies point to an interplay between autophagy and proper lysosomal function [10]. Lysosomes are single-membrane acidic organelles that carry out essential cellular functions, including degradation of intracellular and extracellular material, cellular signaling, energy metabolism and cell death [11]. Lysosomal dysfunction is increasingly recognized as a common mechanism that contributes to the pathogenesis of diverse disorders [12,13,14,15].

The formation of mature lysosomes is a complex process involving several organelles or structures, such as the Golgi apparatus, endosomes, autolysosomes and plasma membrane [16,17,18]. Mature lysosomes have specific membrane-associated proteins, such as LAMP1, and contain several hydrolytic enzymes in their acidic lumen that degrade cytoplasmic and foreign proteins [19,20]. One of the main cellular endopeptidases located in the endosomal/lysosomal compartment is cathepsin D. This aspartic protease is involved in the degradation of many substrates, and its dysfunction leads to the accumulation of nondegraded substrates in the lysosomes [21,22].

Alterations in lysosomal function are produced by the inadequate acidification of the lysosomal lumen, decreased activity of degradative enzymes or the permeabilization of lysosomal membranes, among other factors [23]. Lysosomal dysfunction or defects in the fusion of lysosomes with autophagosomes and endosomes lead to impairment of metabolic, endocytic, phagocytic and autophagic pathways [10].

The function of KEAP1 as an NRF2 repressor and redox sensor has been well-established. However, KEAP1 is not just a repressor of NRF2, and its role in cellular proteostasis has not been fully understood. Here, we show that the loss of KEAP1 promotes the accumulation of nondegradative vesicles that do not contain cathepsin D, the major lysosomal hydrolase. We further demonstrate that KEAP1-deficient cells exhibit autophagic changes and are more sensitive to lysosomal dysfunction.

## 2. Materials and Methods

### 2.1. Cell Culture

We used mouse embryonic fibroblast (MEFs) from wild-type (WT) or KEAP1-deficient (Keap1^KO^) mice that were provided by Dr. Ken Itoh (Center for Advanced Medical Research, Hirosaki University Graduate School of Medicine, Hirosaki Japan). MEF cells were maintained in Dulbecco’s Modified Eagle’s Medium with high glucose, supplemented with 10% heat-inactivated fetal bovine serum (Sigma–Aldrich, Merck KGaA, Darmstadt, Germany, F7524) and 2 mL penicillin–streptomycin (10 U/mL and 100 µg/mL, respectively) (Gibco, Grand Island, NY, USA, SV30010) at 37 °C and 5% CO_2_ in humidity-saturation conditions. The cells were passaged every 24–48 h and used under 12 passages.

### 2.2. Reagent and Chemical Treatments

Cells were grown to 80% confluence for 24 h in 6-/24-/96-well plates. Twenty-four h after seeding, the culture media was replaced with media containing the indicated treatments and the cells were incubated for different times according to the experiment: bafilomycin A1 100 nM (LC Laboratories, Woburn, MA, USA, B1080), rapamycin 1 µM (LC Laboratories, Woburn, MA, USA, R-5000), L-leucyl-L-leucine methyl ester (LLOMe) 1 mM (Sigma–Aldrich, Merck KGaA, Darmstadt, Germany, L7393) and chloroquine (CQ) 50 μM (Sigma–Aldrich, Merck KGaA, Darmstadt, Germany, C6628).

### 2.3. Protein Extraction and Western Blot Analysis

Cell lysis was performed using NP40 lysis buffer 0.5% (*v*/*v*) with Tris–HCl 0.5 M, pH 6.8, and 150 mM NaCl in Milli-Q water, supplemented with protease inhibitor cocktail tablets (cOmplete Mini, EDTA-free, Roche Diagnostics Deutschland GmbH, Mannheim, Germany, No. 11836170001) and phosphatase inhibitor cocktail tablets (PhosSTOP, Roche Diagnostics Deutschland GmbH, Mannheim, Germany,, No. 04906837001). Whole-cell lysate and the nonsoluble fraction were obtained using SB1x 2% (*v*/*v*) SDS, 10% (*v*/*v*) glycerol and 50 mM Tris–HCl, pH 6.8. Protein concentrations were determined with the BCA method, and the samples were diluted to the desired concentration (10–15 µg/lane) with PBS buffer and 5× sample loading buffer (0.025% (*v*/*v*) bromophenol blue, 5% (*v*/*v*) β-mercaptoethanol, 50% (*v*/*v*) glycerol, 0.01 M sodium acetate, pH 5.2, and 250 mM Tris–HCl, pH 6.8). Protein dual color standards (Biorad, Hercules, CA, USA, #1610374) were loaded in one lane per gel.

For protein electrophoresis, samples were loaded in precast gels (Biorad, Hercules, CA, USA, #4561093) and run in 1X TGS buffer (Tris-Glycine-SDS, Fisher Bioreagents, BP1341) at 100 mV. Once finished, the proteins were transferred to a PVDF membrane according to the humidity transference protocol described in [24]. Then, the membranes were blocked (1 h at room temperature) with 10% (*w*/*v*) fat-free milk in Tris-buffered saline (10 mM Tris/HCl, pH 7.5, 150 mM NaCl) containing 0.2% Tween 20 (#P5927, Sigma–Aldrich, Merck KGaA, Darmstadt, Germany) (TBST). The membranes were washed with 1X TBST and incubated overnight at 4 °C with the corresponding primary antibodies: ACTB (#ab49900, Abcam, Cambridge, UK, 1:50,000), CTSC (D-6) (sc-74590, Santa-Cruz Biotechnology, Paso Robles, CA, USA, 1:1000), CSTD (D-7) (sc-377299, Santa-Cruz Biotechnology, Paso Robles, CA, USA, 1:1000), EEA1 (#3288, Cell Signaling Technologies, Danvers, MA, USA, 1:1000), LAMP1 (#ab_657536, Abcam, Cambridge, UK, 1:1000), LC3 (#L7543, Sigma–Aldrich, Merck KGaA, Darmstadt, Germany, 1:5000), p62/SQSTM1 (#H00008878-MO1, Abnova, Taipei, Taiwan, 1:1000), RAB7 (D95F2) (#9367, Cell Signaling Technologies, Danvers, MA, USA, 1:1000), p-p70S6K (Thr389, #9205 Cell Signaling Technologies, Danvers, MA, USA, 1:1000), p70S6K (#9202, Cell Signaling Technologies, Danvers, MA, USA, 1:1000), p-TFEB (Ser142, ABE1971, Sigma–Aldrich, Merck KGaA, Darmstadt, Germany, 1:500), α-tubulin (#3873S, Cell Signaling Technologies, Danvers, MA, USA, 1:1000) and ubiquitin (P4D1) (sc-8017, Santa-Cruz Biotechnology, Paso Robles, CA, USA,1:1000). After several washing steps in 1X TBST, the membranes were incubated with their respective HRP-conjugated secondary antibodies (1:10,000) (#170-6515 and #170-5047, Biorad, Hercules, CA, USA), for rabbit and mouse antibodies, respectively) for 1 h at room temperature. Western blot images were analyzed with Image J software (National Institute of Health, Bethesda, MD, USA).

### 2.4. Immunofluorescence

Cells were seeded in 96-well black plates. Treatments were performed as described previously. The fixation step protocol was followed [25]. After permeabilization, the cells were incubated with primary antibodies against CSTD (D-7) (sc-377299, Santa-Cruz Biotechnology, Paso Robles, CA, USA, 1:200), LAMP1 (H4A3) (sc-20011, Santa-Cruz Biotechnology, Paso Robles, CA, USA, 1:200) and ubiquitin (P4D1) (sc-8017, Santa-Cruz Biotechnology, Paso Robles, CA, USA, 1:200) for 1–2 h at room temperature and then incubated with Alexa Fluor^®^ 568 (A11004)- or 488 (A11008)-conjugated secondary antibodies (Thermo Fisher Scientific, Waltham, MA, USA) for 1 h at room temperature. Image quantification was conducted with Image J software (National Institute of Health, Bethesda, MD, USA).

### 2.5. Long-Lived Protein Degradation Assay

A long-lived protein degradation assay was performed using a pulse-chase experiment. Cells were incubated in the presence of ^14^C Valine (Perkin-Elmer, Waltham, MA, USA, NEC291EU050UC) at 0.2 µC/mL in complete medium for 24 h (pulse). The pre-chase was performed by incubating cells in the presence of 10 nM of “cold” Valine (Sigma–Aldrich, Merck KGaA, Darmstadt, Germany, V513). Chase steps were performed with treatment diluted in complete medium supplemented with 10 nM L-valine. Three different extracts were obtained and measured with a scintillation counter. A fully detailed protocol is available in [26].

### 2.6. Protease Activity Assays

To assess cathepsin D activity, we performed a fluorimetric assay. For lysis of the cellular pellet, we used cathepsin buffer: sodium acetate 50 mM, pH 5.5, NaCl 0.1 M, EDTA 1 mM, 0.2% (*v*/*v*) Triton-X100. The pellet was resuspended by gentle pipetting and then placed in a rotational shaker at 4 °C for 30 min. The cell debris was precipitated by centrifugation at 21,694× *g*. The activity of the enzyme was determined from the consumption of cathepsin D fluorigenic substrate (BML P145-0001), Ex.: 360 nm, Em.: 465 nm, in the presence of leupeptin for other cathepsin inhibitions and compared with the same reaction in the presence of pepstatin A, an aspartic protease inhibitor that inhibits cathepsin D, among other substances.

### 2.7. Flow Cytometry

For the detection of acidic vesicles, we performed a cytometry assay. The cells were plated for 24 h prior to the experiment and then treated for 30 min with LysoSensor green DND189 (Invitrogen, Waltham, MA, USA, L7535) and LysoTracker (LTR, Invitrogen, Waltham, MA, USA, L7528) probes in the last 15 min of treatment. Then, the cells were trypsinized and transferred to cytometry tubes with culture media.

### 2.8. Electron Microscopy

Cells were treated in 6-well plates for each condition. Cultured media and/or treatments were removed, and the cells were washed once with PBS. The cells were removed from the plate with 200 μL of trypsin and then collected in a 1.5 mL microcentrifuge tube for centrifugation at 232× *g* 5′, 4 °C. After centrifugation, the cell pellet was washed with PBS without moving the pellet. The cells were fixed with glutaraldehyde 2.5% for 2 h at 4 °C. Then, the cells were washed with sodium cacodylate 0.1 M, pH 7.4, three times, followed by centrifugation at 232× *g* and 4 °C between each wash. The samples were maintained in sodium cacodylate 0.1 M, pH 7.4, at 4 °C for a short time prior to analysis by an electron microscopy service (CITIUS, University of Seville, Seville, Spain).

### 2.9. RT-PCR

A two-step technique was used. First, RNA extraction was performed with an RNAeasy mini-kit (Qiagen, Hilden, Germany, 74104) and, once extracted, RNA was purified using DNAseI (Sigma–Aldrich, Merck KGaA, Darmstadt, Germany, AMPD1-1KT) and quantified using a Qubit Fluorimeter with a Quibit RNA BR assay kit (Invitrogen, Waltham, MA, USA, Q10210). cDNA synthesis was performed with 1 ng of RNA with a NZYTECH First-Strand cDNA synthesis kit (MB12501) using equal concentrations of RNA template. qPCR was performed with Sybr Green reagents with 1 µL of cDNA using the following primers: TFEB (FW: 5′-AAGCAGCTCTGGCTCCGCAT-3′/RV: 5′-CGGCATTTGCTCAGGCTCAG-3′), KEAP1 (FW: 5′-GGGCTT TGACGGGACTAACC-3′/RV: 5′-ATCCGCCACTCATTCCTCTCT-3′) and GAPDH (FW: 5′-AACTTTGGCATTGTGGAAG-3′/RV: 5′-ACACATTGGGGGTAGAAA-3′).

### 2.10. Statistical Analysis

Data are presented as means ± SD (standard deviation). Statistical assessments were carried out using Prism 6 software (GraphPad Software, San Diego, CA, USA). For the statistical comparison of two groups, we performed two-tailed Student’s *t*-tests. For statistical comparison of multiple groups, we performed two-way ANOVA following by Tukey’s or Sidak tests.

## 3. Results

### 3.1. KEAP1 Deficiency Enhanced the Number of Acidic Vesicles

**Figure 1:**
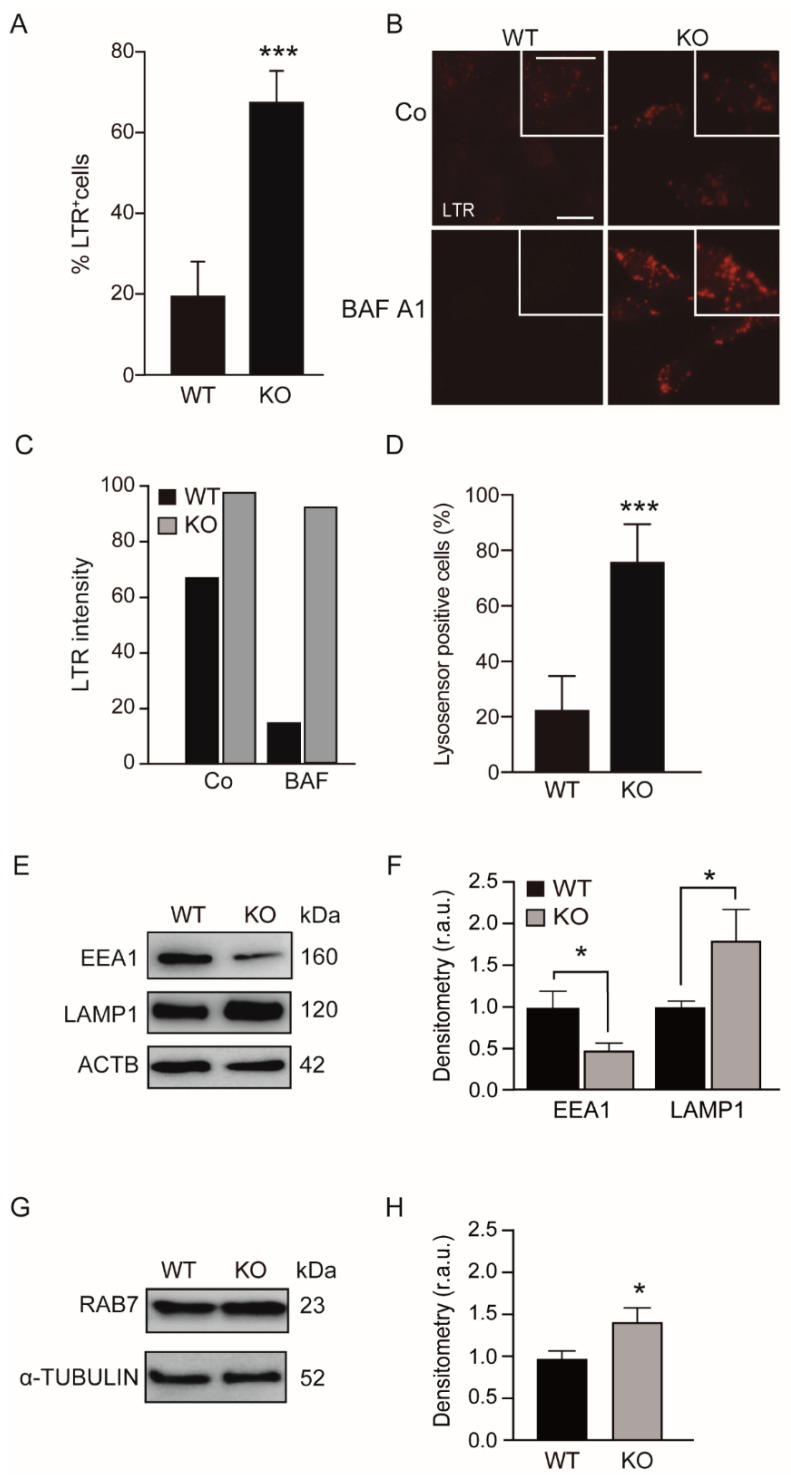
The loss of KEAP1 induces the accumulation of acidic vesicles. WT and Keap1^KO^ mouse embryonic fibroblasts (MEFs) were cultured in control conditions (Co) (**A,D**–**H**) or incubated with 100 nM bafilomycin (Baf. A1) for 4 h (**B**,**C**) and stained with LysoTracker red (LTR) (**A**) and LysoSensor (**D**) probes, as described in Section 2. Next, the cells were analyzed with flow cytometry (**A**,**D**) and fluorescence microscopy (**B**). Representative images show LTR-positive cells, (*** *p* < 0.001 vs. WT cells) (**B**) and the LTR intensity was quantified as shown in (**C**). The scale bar represents 10 μm. **(D**) The graph shows the percentage of LysoSensor-positive cells (*** *p* < 0.001 vs. WT cells). (**E**,**G**) Cell lysates from WT and Keap1^KO^ MEFs were analyzed by Western blot using anti-EEA1, RAB7 and LAMP1 antibodies. α-tubulin and ACTB were used as a loading control. (**F**,**H**) Densitometry was employed to quantify the abundance of EEA1 and LAMP1 normalized to ACTB (**F**) and RAB7 normalized to α-tubulin (**H**) (* *p* < 0.05). All data are the means ± SD of at least three independent experiments and they were analyzed using Student’s *t*-test. r.a.u, relative arbitrary units.

First, we examined whether KEAP1 deficiency affects the endosomal-lysosomal pathway using LysoTracker red (LTR), a fluorescent dye that preferentially accumulates in acidic vesicles, such as lysosomes and endosomes. We found that Keap1 deficiency (Figure S1a) enhanced the number of acidic vesicles (Figure 1A). Surprisingly, when we used Baf. A1, an inhibitor of the vacuolar proton pump, to increase the intralysosomal pH, we observed similar fluorescence intensity of LTR staining in the Keap1^KO^ cells (Figure 1B,C). These results contrast with the WT MEFs treated with Baf. A1, which showed a dramatic reduction in LTR intensity. Therefore, KEAP1-deficient cells are less sensitive to Baf. A1.

We used the LysoSensor dye to monitor the acidification levels of the autolysosomal and/or lysosomal compartments [27]. Flow cytometry analysis showed that the lysosomal compartments of KEAP1-deficient cells (Figure 1D) were more acidic than those of the WT cells. Next, we analyzed the levels of endosomal-lysosomal proteins using Western blotting and we observed elevated levels of the lysosome-associated membrane protein 1 (LAMP1) when KEAP1 was missing (Figure 1E,F). Moreover, we found increased levels of the endosomal membrane marker RAB7 in Keap1^KO^ cells. In contrast, the levels of the early endosome protein marker EEA1 were markedly reduced in Keap1^KO^ cells.

### 3.2. The mTOR Pathway Is Activated in KEAP1-Deficient Cells

Next, we analyzed whether the accumulation of lysosomal-endosomal vesicles observed in Keap1^KO^ cells was due to the induction of lysosomal biogenesis.

The mTORC1-TFEB axis controls lysosomal biogenesis in response to nutrient availability and growth factors, among other influences [28]. TFEB is a transcription factor that activates autophagic and lysosomal genes, thereby increasing the number of lysosomes. Under normal conditions, TFEB is phosphorylated by mTOR and sequestered in the cytoplasm. Under starvation, mTOR inhibition induces TFEB nuclear translocation by reducing TFEB phosphorylation [29,30].

**Figure 2:**
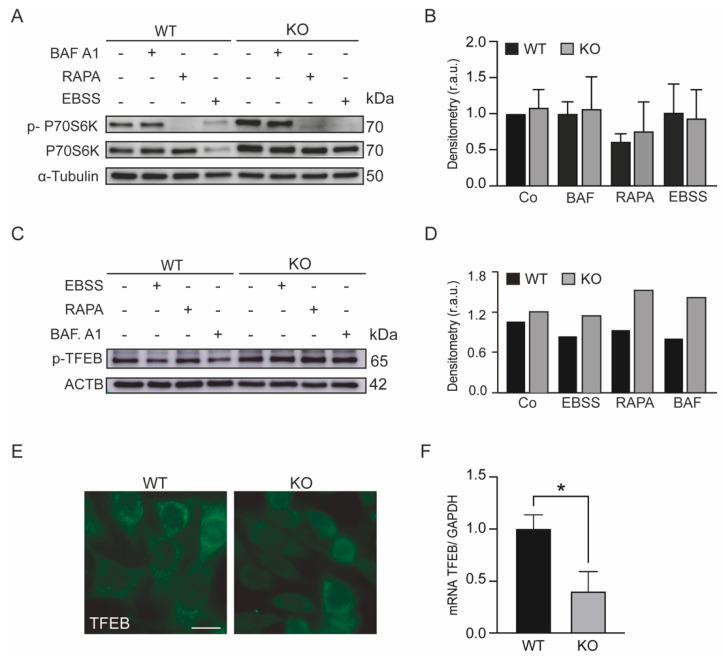
KEAP1 deficiency activates the mTOR pathway. (**A**,**B**) WT and Keap1^KO^ mouse embryonic fibroblasts (MEFs) were cultured in control conditions and treated with 1 µM rapamycin (RAPA) or incubated with nutrient-free (EBSS) medium for 4 h. (**A**) p–p70S6K and p70S6K were assessed by immunoblotting. α-tubulin was used as a loading control. (**B**) Densitometry was employed to quantify the abundance of p–p70S6K normalized to α-tubulin. (**C**,**D**) WT and Keap1^KO^ MEFs were cultured in control conditions, incubated with EBSS medium, treated with 1 µM RAPA or incubated with 100 nM Baf. A1 for 4 h. (**C**) p-TFEB was assessed by immunoblotting. ACTB was used as a loading control. (**D**) Densitometry was employed to quantify the abundance of p-TFEB normalized to ACTB. (**E**) Representative immunofluorescence images of cells immunolabeled with TFEB. The scale bar represents 10 μm. (**F**) WT and Keap1^KO^ MEF RNA were extracted, and real-time quantitative PCR was performed for the TFEB gene. GAPDH was used as an endogenous control of gene expression. The histogram shows the means ± SD from at least three independent experiments and they were analyzed using Student’s *t*-test (* *p* < 0.05).

First, we assessed mTORC1 activity in KEAP1-deficient cells by analyzing the phosphorylation levels of p70S60K (RPS6KB1), which is a well-established marker of mTORC1 activity. Figure 2A,B show that the loss of KEAP1 increased the relative p-p70S6K levels, indicating increased mTORC1 activity.

Next, we analyzed the phosphorylation levels of TFEB using Western blotting and its localization with immunofluorescence. We found that KEAP1-deficient cells showed similar levels of p-TFEB (Ser 142) compared to WT cells, remaining sequestered in the cytosol (Figure 2C–E). Moreover, the mRNA expression level of TFEB was decreased in Keap1^KO^ cells (Figure 2F). Together, these results indicated that the loss of KEAP1 does not induce lysosomal biogenesis through the mTOR/TFEB pathway.

### 3.3. Loss of KEAP1 Led to Decreased Cathepsin D Activity

**Figure 3:**
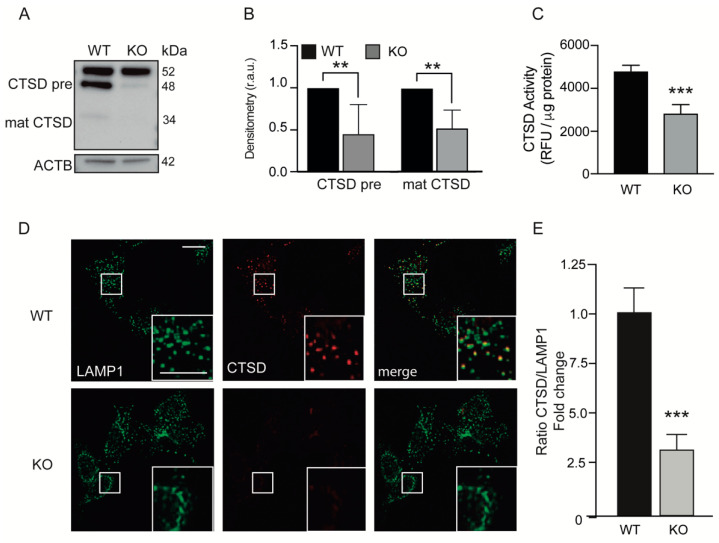
KEAP1-deficient cells show LAMP1-labeled vesicles with reduced CTSD activity. (**A**) Cell lysates from WT and Keap1^KO^ MEFs were analyzed with Western blotting using anti-CSTD antibody. ACTB was used as a loading control. (**B**) Densitometry was employed to quantify the abundance of different isoforms of CTSD normalized to ACTB (** *p* < 0.01 vs. WT cells). (**C**) The graph shows the CTSD activity in relative fluorescence units (r.f.u.) per microgram of proteins (*** *p* < 0.001 vs. WT cells). (**D**) Representative immunofluorescence images of cells costained with LAMP1 and CTSD. The scale bar represents 10 μm. (**E**) The graph shows the ratio of CTSD/LAMP1 (*** *p* < 0.001 vs. WT cells). All data are the means ± SD of at least three independent experiments and they were analyzed using Student’s *t*-test.

Lysosomes contain hydrolytic enzymes that degrade all types of molecules. Cathepsins are the most abundant lysosomal proteases. Therefore, we studied the effect of KEAP1 deficiency on lysosomal content by analyzing the cathepsin C (CTSC) and D (CTSD) protein levels, and we observed decreased proteolytic cleavage of CTSD from the immature and intermediate forms (52–48 kDa) into the mature form (33 kDa) in Keap1^KO^ cells (Figure 3A,B). A reduction in CTSC protein levels was also observed in Keap1^KO^ cells (Figure S1b,c). We confirmed these results by analyzing the level of CTSD using immunofluorescence, and we observed that KEAP1-deficient cells showed reduced CTSD staining (Figure 3D). The ratio of CTSD/LAMP1 was robustly reduced in KEAP1-deficient cells (Figure 3E). Moreover, we analyzed whether the loss of KEAP1 modulates CTSD activity by performing a CTSD fluorometric activity assay and we found that Keap1^KO^ cells showed reduced CTSD activity compared with control cells (Figure 3C).

Therefore, our data suggest that there is a link between CTSD and KEAP1 proteins.

### 3.4. KEAP1 Deficiency Induces Autophagic Changes

**Figure 4:**
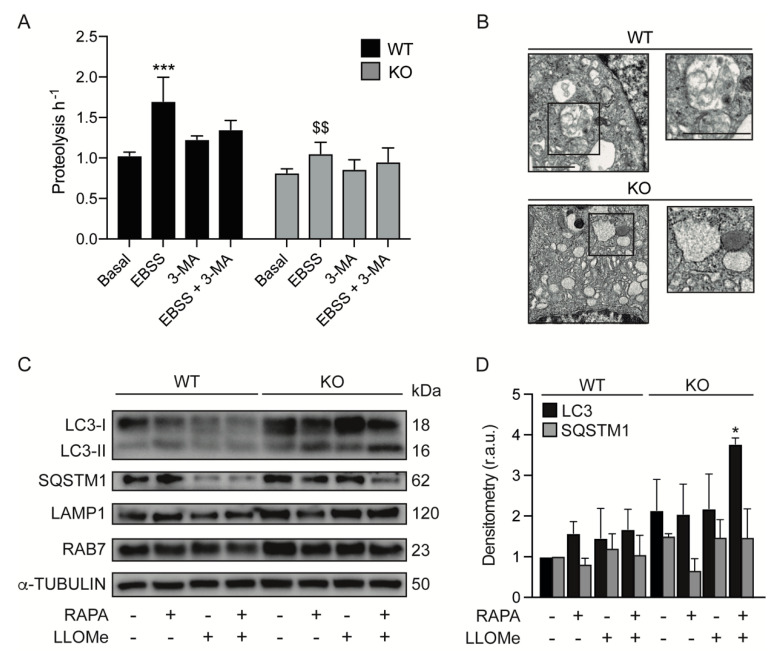
KEAP1 knockout induces autophagic changes. (**A**) WT and Keap1^KO^ MEFs were cultured under basal conditions or incubated with nutrient-free (EBSS) medium alone or in combination with 10 mM 3–methyladenine (3–MA) for 2 h. Long-lived protein degradation was determined with a pulse-chase assay, as indicated in Section 2. Data are the means ± SD of at least three independent experiments and they were analyzed using two-way ANOVA following by Tukey’s multiple comparisons test (*** *p* < 0.001 vs. basal condition and $$ *p* < 0.01 vs. WT cells). (**B**) Representative TEM micrographs of WT and Keap1^KO^ MEFs show different structures. The scale bar represents 2 μm. (**C**) WT and Keap1^KO^ MEFs were cultured in control conditions or incubated with 1 mM of LLOMe for 1 h followed by a washout step of 4 h. Additionally, one condition involved treatment with 1 μM rapamycin in the last 2 h of the washout. LC3, p62, LAMP1 and RAB7 were assessed with immunoblotting. α-tubulin was used as a loading control. (**D**) Densitometry was employed to quantify the abundance of the indicated proteins normalized to α-tubulin. Data are the means ± SD of at least three independent experiments and they were analyzed using two-way ANOVA following by Sidak’s multiple comparisons test (* *p* < 0.05 vs. WT cells).

Next, we investigated whether the absence of active CTSC and CTSD reduces the degradative activity (degradation rate) of KEAP1-deficient cells. Our results showed that, despite the elevated number of acidic vesicles, Keap1^KO^ cells showed no significant changes in lysosomal degradation capacity compared to control cells (Figure 4A). However, the induction of autophagy during EBSS incubation did not increase the degradation rate of lysosomes in KEAP1-deficient cells. Electron microscopy analysis revealed that Keap1^KO^ cells showed more vesicles in the cytoplasm than control cells; however, these compartments did not show electron-dense material (Figure 4B).

Lysosomes play an essential role in the autophagic pathway. In fact, in the last stage of autophagy, autophagosomes fuse with lysosomes and the content of the autophagosomes is eliminated by lysosomal degradative enzymes [31]. Therefore, defects in lysosomal function affect autophagy. We used LLOMe, which is converted into a membranolytic polymeric form in the lysosome lumen by lysosomal hydrolases that permeabilize the lysosomal membrane, altering lysosomal function [32]. The analysis of the LC3 and p62 protein levels following the treatment with RAPA and LLOMe showed that there was an accumulation of both proteins in KEAP1-deficient cells (Figure 4C,D). This accumulation was more obvious when the lysosome was damaged by LLOMe treatment.

### 3.5. Lysosomes of Keap1^KO^ Cells Are More Sensitive to Damage

**Figure 5:**
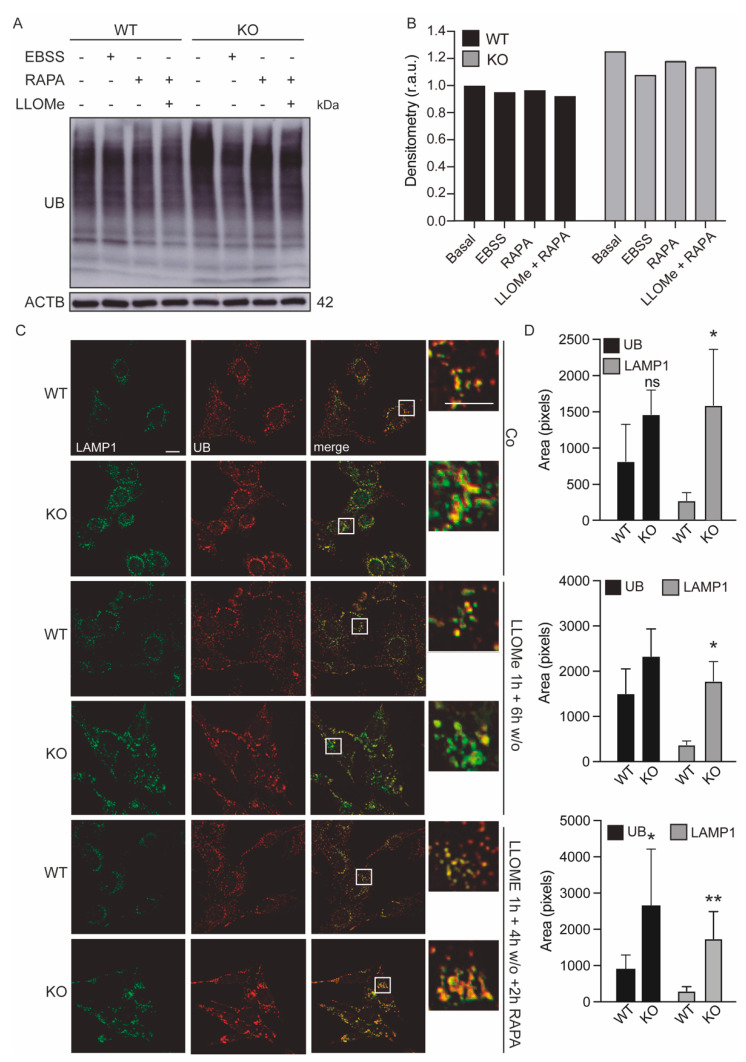
LAMP1–labeled structures of Keap1^KO^ cells are more sensitive to damage. (**A**,**B**) WT and Keap1^KO^ MEFs were cultured in control conditions, incubated with nutrient-free (EBSS) medium or treated with 1 μM rapamycin (RAPA) alone or in combination with 1 mM LLOMe. Ubiquitin was assessed with immunoblotting. ACTB was used as a loading control. Densitometry was employed to quantify the abundance of ubiquitin normalized to ACTB. (**C**,**D**) WT and Keap1^KO^ MEFs were cultured in control conditions or incubated with 1 mM LLOMe for 1 h followed by a washout step, and 1 μM rapamycin was added to one treatment in the last 2 h of washout. (**C**) Representative immunofluorescence images of cells costained with LAMP1 and UB antibodies. The scale bar represents 10 μm. (**D**) The areas of ubiquitin-positive vesicles and LAMP1–positive vesicles were quantified and analyzed using two-way ANOVA following by Sidak’s multiple comparisons test. * *p* < 0.05, ** *p* < 0.01 vs. WT cells.

To further investigate the effect of KEAP1 deficiency on lysosome function, we treated the cells with LLOMe and we observed that Keap1^KO^ cells were more sensitive to LLOMe after 1 h of treatment and had diminished recovery from lysosomal damage upon washout (Figure 5C,D). Moreover, the analysis of the images showed that LLOMe treatment enhanced the number of peripheral LAMP1-positive vesicles in KEAP1-deficient cells compared to control. We confirmed these results with Western blot analysis of ubiquitinated protein levels and we observed that Keap1^KO^ cells showed increased levels of ubiquitinated protein levels after LLOMe treatment with and without washout (Figure 5A,B and Figure S2a,b).

## 4. Discussion

A close relationship between KEAP1 and autophagy has been demonstrated. In fact, the loss of KEAP1 promotes the accumulation of ubiquitin aggregates and defective autophagy activation [9]. However, there is no evidence that KEAP1 is involved in endocytic-lysosomal pathways. The present study demonstrated that the loss of KEAP1 causes the accumulation of LAMP1-labeled nondegradative vesicles characterized by reduced protein levels and proteolytic activity of cathepsin D.

Lysosomes are degradative organelles that play important roles in multiple processes, such as degradative pathways and apoptotic and metabolic pathways [33]. Mature lysosomes are characterized by an acidic environment and they contain high levels of lysosomal membrane proteins and lysosomal hydrolases [19]. In fact, LAMP1 is commonly used as a lysosome marker and LAMP1-positive vesicles are defined as lysosomes. Our data showed that the loss of KEAP1 promotes the accumulation of acidic vesicles and that these vesicles are labeled by LAMP1. However, LAMP1 is also present in many nonlysosomal compartments [34]. Therefore, we studied the expression levels of several endocytic proteins and we found that KEAP1-depleted cells showed increased levels of the late endosome marker RAB7. In contrast, the levels of early endosome protein 1 (EEA1) were diminished compared to those in control cells.

Lysosomal biogenesis has not been fully elucidated. Lysosomes can be formed from endosomes. In fact, late endosomes may lose their endosomal membrane markers, such as RAB7, and acquire LAMP1 or LAMP2 markers as they are converted into lysosomes. Moreover, lysosomes are also regenerated from autolysosomes in a process called autophagy lysosome reformation (ARL) [17]. TFEB is the major activator of lysosomal biogenesis [35]. It translocates to the nucleus and promotes the expression of multiple lysosomal and autophagy genes. Although TFEB upregulates the expression of NRF2 and its downstream genes [36], it does not modulate the mRNA levels of KEAP1 [37]. However, our data show that the loss of KEAP1 significantly decreased the mRNA levels of TFEB. Paradoxically, there were no changes in the phosphorylation levels of TFEB in KEAP1-deficient cells as compared to WT cells. Therefore, loss of KEAP1 does not induce lysosomal biogenesis via mTORC1/TFEB, so the accumulation of vesicles could be mediated through another pathway.

The degradative capacity of the lysosomes depends on their hydrolytic enzymes. An acidic environment is crucial for proteolytic processing and the maturation of these hydrolases [38]. Our data demonstrated that loss of KEAP1 affects the maturation and enzymatic functions of cathepsin D. In fact, our results reveal that KEAP1-depleted cells show a significant number of LAMP1-labeled compartments that do not contain lysosomal D hydrolases. These results are consistent with a recent study performed on neurons that identified LAMP1-positive organelles that do not contain lysosomal hydrolase, which represent nondegradative organelles [39]. Therefore, KEAP1 deficiency seems to enhance the number of nondegradative structures.

Defective lysosomal proteolysis may influence cell signaling pathways, such as autophagy. Studies have demonstrated that KEAP1 levels are reduced by autophagy [8,40,41,42]. In fact, the levels of KEAP1 were increased in Atg7-knockout mice and reduced upon starvation [42]. Our results showed increased LC3, p62 and ubiquitin protein levels in KEAP1-deficient cells.

Finally, several studies have revealed a link between lysosomal position and function [43,44]. Peripheral lysosomes display less degradation than with perinuclear lysosomes. However, another study of neurons suggested that changes in the distribution, density and trafficking of LAMP1-positive vesicles do not represent degradative lysosomes [45]. Analysis of the effect of LLOMe treatment in KEAP1-deficient cells showed that LLOMe treatment enhanced the number of peripheral LAMP1-labeled vesicles in Keap1-deficient cells. Moreover, the loss of KEAP1 increased the sensitivity of these cells to lysosomal alteration compared to control cells.

The function of KEAP1 as an NRF2 repressor has been well-established. However, another noncanonical function of KEAP1 exists that should be studied. Although those pathways were not studied in our experimental model, our data suggest that KEAP1 has a role in the endosomal-lysosomal pathway and it is important for lysosomal maturation. However, it is not clear how KEAP1 affects cathepsin D activity and promotes the accumulation of nondegradative vesicles. The challenge in the future is to determine the mechanism that regulates the endosomal-lysosomal pathway in KEAP1-deficient cells.

## Data Availability

Data are contained within the article and Supplementary Materials.

## Supplementary Materials

The following are available online at https://www.mdpi.com/article/10.3390/antiox11071398/s1, Figure S1: KEAP1-deficient cells show reduced CTSC protein levels. Figure S2: KEAP1 deficiency induces ubiquitinated protein accumulation.
