# Supplementary material for: Loss of KEAP1 Causes an Accumulation of Nondegradative Organelles
Source: Antioxidants (Basel). 2022 Jul 19;11(7):1398. doi: 10.3390/antiox11071398 (PMC9311848; doi:10.3390/antiox11071398)
Supplement: Supplementary file 1 [file antioxidants-11-01398-s001.zip › antioxidants-1795660-supplementary.pdf]

antioxidants

Supplementary material to:

Loss of KEAP1 Causes an Accumulation of Nondegradative Organelles

Elisabet Uribe-Carretero [1,2,3], Guadalupe Martinez-Chacón [1,2,3], Sokhna M. S. Yakhine-Diop [2], Gema Duque-González [1], Mario Rodríguez-Arribas [1], Eva Alegre-Cortés [1,2,3], Marta Paredes-Barquero [1,2,3], Saray Canales-Cortés [1,2,3], Elisa Pizarro-Estrella [1], Antonio Cuadrado [2,4,5,6], Rosa Ana González-Polo [1,2,3,*], José M. Fuentes [1,2,3,*] and Mireia Niso-Santano [1,2,3,*]

1 Departamento de Bioquímica y Biología Molecular y Genética, Facultad de Enfermería y Terapia Ocupacional, Universidad de Extremadura, 10003 Cáceres, Spain; euribec@unex.es (E.U.-C.); gmartinezchacon@hotmail.com (G.M.-C.); geduqueg@unex.es (G.D.-G.); mariora@unex.es (M.R.-A.); evalegrec@unex.es (E.A.-C.); martapb@unex.es (M.P.-B.); sacanalesc@unex.es (S.C.-C.); osyris_tng@hotmail.com (E.P.-E.)
2 Centro de Investigación Biomédica en Red en Enfermedades Neurodegenerativas (CIBERNED), 28029 Madrid, Spain; smsyakhinediop@unex.es (S.M.S.Y.-D.); antonio.cuadrado@uam.es (A.C.)
3 Instituto Universitario de Investigación Biosanitaria de Extremadura (INUBE), 10003 Cáceres, Spain
4 Departamento de Bioquímica, Facultad de Medicina, Universidad Autónoma de Madrid (UAM), 28049 Madrid, Spain
5 Instituto de Investigaciones Biomédicas Alberto Sols (CSIC-UAM), 28029 Madrid, Spain
6 Instituto de Investigación Sanitaria La Paz (IdiPaz), 28029 Madrid, Spain
* Correspondence: rosapolo@unex.es (R.A.G.-P.); jfuentes@unex.es (J.M.F.); mnisosan@unex.es (M.N.-S.)

Citation: Uribe-Carretero, E.; Martinez-Chacón, G.; Yakhine-Diop, S.M.S.; Duque-González, G.; Rodríguez-Arribas, M.; Alegre-Cortés, E.; Paredes-Barquero, M.; Canales-Cortés, S.; Pizarro-Estrella, E.; Cuadrado, A.; et al. Loss of KEAP1 Causes an Accumulation of Nondegradative Organelles. *Antioxidants* **2022**, *11*, 1398. https://doi.org/10.3390/antiox11071398

Academic Editor: Young-Sam Keum

Received: 24 June 2022
Accepted: 15 July 2022
Published: 19 July 2022

Publisher's Note: MDPI stays neutral with regard to jurisdictional claims in published maps and institutional affiliations.

Copyright: © 2022 by the authors. Licensee MDPI, Basel, Switzerland. This article is an open access article distributed under the terms and conditions of the Creative Commons Attribution (CC BY) license (https://creativecommons.org/licenses/by/4.0/).

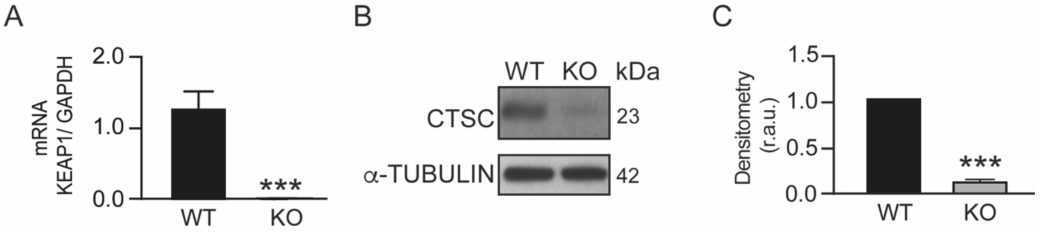

Figure S1. KEAP1-deficient cells show LAMP1-labeled vesicles with reduced CTSC protein levels. (**A**) *WT* and *Keap1^{KO}* MEFs RNA was extracted, and real-time quantitative PCR was performed for KEAP1 gene. GAPDH was used as an endogenous control of gene expression. The histogram shows the mean ± SD of at least three independent experiments. (**B**) Cell lysates from *WT* and *Keap1^{KO}* MEFs were analyzed by western blot using anti-CSTC antibody. α–tubulin was used as a loading control. (**C**) Densitometry was employed to quantify the abundance of CTSC. r.a.u means relative arbitrary units. All data are the mean ± of at least three independent experiments and they were compared by Student's t-test (***p< 0.001 versus *WT* cells).

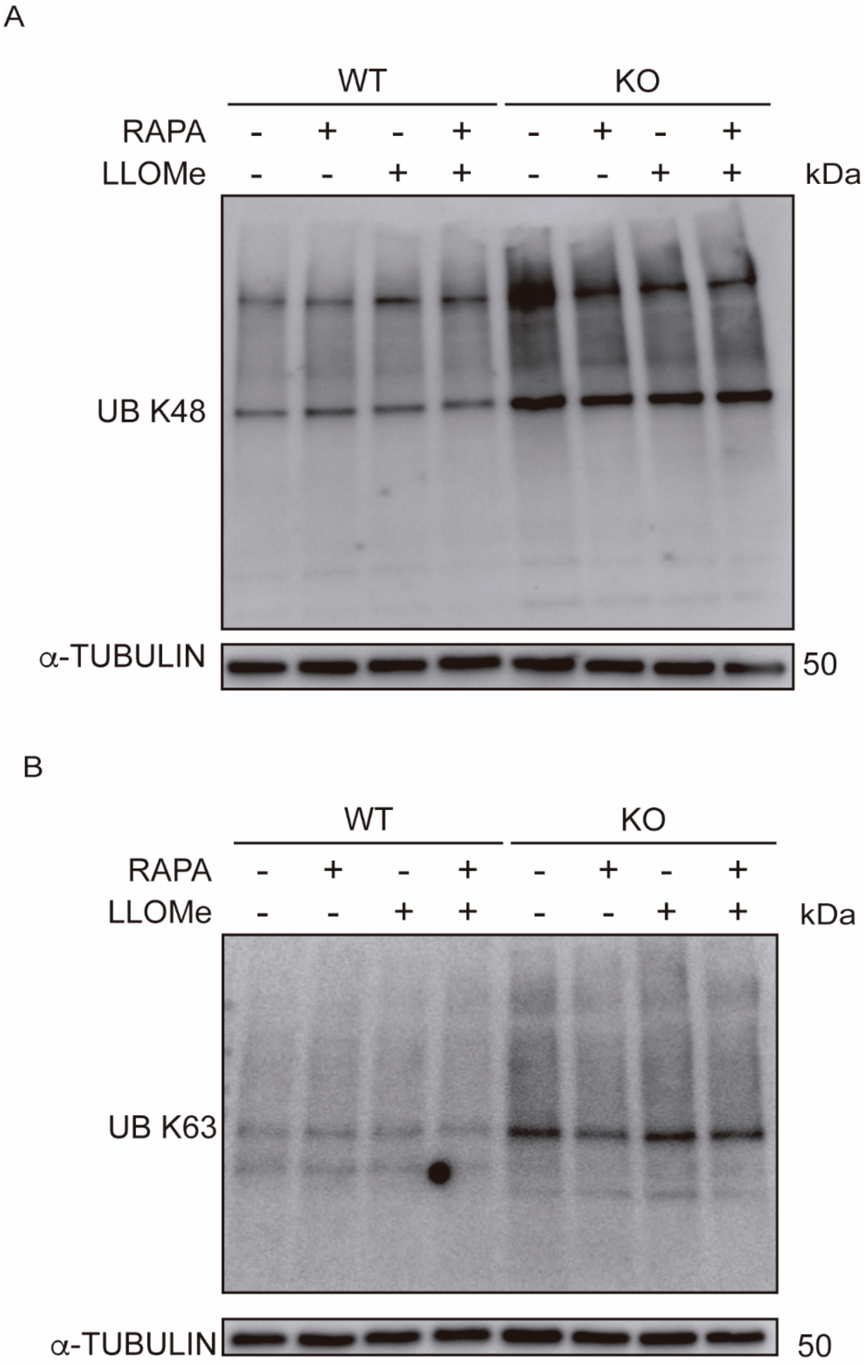

Figure S2. KEAP1 deficiency induces ubiquitinated protein accumulation. (**A**,**B**) WT and Keap1^{KO} MEFs were cultured in control conditions, incubated with 1mM LLOMe or treated with 1 mM rapamycin (RAPA) alone or in combination with 1 mM LLOMe. UB K63 and UB K48 were assessed by immunoblotting. α-tubulin was used as a loading control.
